# Pre-Emptive Treatment of Lidocaine Attenuates Neuropathic Pain and Reduces Pain-Related Biochemical Markers in the Rat Cuneate Nucleus in Median Nerve Chronic Constriction Injury Model

**DOI:** 10.1155/2012/921405

**Published:** 2011-11-24

**Authors:** Chi-Te Lin, Yi-Ju Tsai, Hsin-Ying Wang, Seu-Hwa Chen, Tzu-Yu Lin, June-Horng Lue

**Affiliations:** ^1^Department of Anatomy and Cell Biology, College of Medicine, National Taiwan University, Taipei 10018, Taiwan; ^2^School of Medicine, College of Medicine, Fu Jen Catholic University, Taipei 24205, Taiwan; ^3^Department of Anatomy, Taipei Medical College, Taipei 100, Taiwan

## Abstract

This study investigates the effects of lidocaine pre-emptive treatment on neuropathic pain behavior, injury discharges of nerves, neuropeptide Y (NPY) and c-Fos expression in the cuneate nucleus (CN) after median nerve chronic constriction injury (CCI). Behavior tests demonstrated that the pre-emptive lidocaine treatment dose dependently delayed and attenuated the development of mechanical allodynia within a 28-day period. Electrophysiological recording was used to examine the changes in injury discharges of the nerves. An increase in frequency of injury discharges was observed and peaked at postelectrical stimulation stage in the presaline group, which was suppressed by lidocaine pre-emptive treatment in a dose-dependent manner. Lidocaine pretreatment also reduced the number of injury-induced NPY-like immunoreactive (NPY-LI) fibers and c-Fos-LI neurons within the CN in a dose-dependent manner. Furthermore, the mean number of c-Fos-LI neurons in the CN was significantly correlated to the NPY reduction level and the sign of mechanical allodynia following CCI.

## 1. Introduction

Pre-emptive analgesia is broadly used in clinical practice for relieving postoperation pain and preventing the subsequent development of chronic neuropathic pain after surgery [1, 2]. In chronic constriction injuries (CCIs) of rat sciatic nerves [3], neuropathic pain behavior was also relieved by pre-emptive treatment of MK-801 [4], nociceptin [5], or lidocaine [6], but little is known about the effect of pre-emptive analgesia on neuropathic pain behavior after median nerve CCI. Attenuating ectopic discharges, originating from the damaged nerves [7, 8] and/or their dorsal root ganglia (DRG) [9], were considered to be one of the pre-emptive analgesia mechanisms to relieve neuropathic pain. Topical or systemic application of local anesthetics has been reported to attenuate ectopic discharges [9, 10]. Clinical studies have also indicated that neuropathic pain is alleviated by application of local anesthetics to the painful target areas [11, 12]. Lidocaine is a local anesthetic that produces a transient analgesic effect in humans affected by neuropathic and postoperative pain [13, 14]. Local pretreatment of lidocaine effectively suppresses injury discharges induced by median nerve transection (MNT) [15], but lack of evidence regarding the median nerve CCI model.

Injury to median nerve, neuropeptide Y-like immunoreactive (NPY-LI) fibers are dramatically induced in the lesion side cuneate nucleus (CN), but not detected in the intact side [16]. Furthermore, given an electrical stimulation to the injured median nerve, c-Fos-like immunoreactive (c-Fos-LI) cells are detected only in the ipsilateral CN [17, 18]. The expression of Fos, which is the protein product of the immediate-early proto-oncogene c-fos, has been accepted as a neural marker of pain [19, 20]. pre-emptive analgesia treatment with lidocaine [21] effectively suppresses c-Fos expression in the spinal cord after peripheral nerve injury. Furthermore, the expression of c-Fos is modulated by NPY and is considered to be involved in neuropathic pain [22]. We previously demonstrated that lidocaine pre-treatment dose-dependently suppressed injury discharges to reduce NPY expression in the CN, which in turn significantly attenuated c-Fos expression after MNT along with electrical stimulation [15]. However, there is still only very little of behavioral evidence to support the effect of pre-emptive treatment on neuropathic pain relief after median nerve CCI.

In this study, we wanted to examine whether a single topical application of lidocaine prior to median nerves undergoing CCI would influence the development of neuropathic pain and ectopic discharges after CCI. Furthermore, morphological changes in NPY and c-Fos expression in the CN were examined to evaluate whether their expression levels correlated with the degree of mechanical allodynia.

## 2. Materials and Methods

The experiments were inspected and approved by the National Science Council Committee and the Animal Center Committee, College of Medicine, National Taiwan University, Taiwan (IACUCA Approval no. 20030114 and no. 20080267). Ethical guidelines from the International Association for the Study of Pain [23] were followed in the use of animals. Animals were housed under approved circumstances with a 12/12 h light/dark cycle with food and water available *ad libitum*.

### 2.1. Chronic Constriction Injury (CCI) Operation

Thirty-three male Sprague-Dawley rats (175–200 g) purchased from BioLASCO (Taiwan) were randomly divided into a control group (sham operation, median nerve exposure without injury, *n* = 3), and pretreated with saline (presaline, *n* = 10), 1% lidocaine (pre-1% lido, *n* = 10) and 5% lidocaine (pre-5% lido, *n* = 10) groups along with CCI. Under chloral hydrate anesthesia (31.5 mg/100 g body weight, i.p.), the above-mentioned pretreated groups alternatively underwent unilateral (*n* = 5) or bilateral (*n* = 5) median nerve CCI operations, the nerves were carefully separated from the surrounding tissue at the level of the elbow immediately proximal to entering between the two heads of the pronator teres muscle. Saline and various concentrations of lidocaine (Sigma, St. Louis, Mo, USA) were applied topically to the exposed median nerves (100 *μ*L) at 15 min prior to CCI. Fifteen minutes after the application of the saline or lidocaine, four ligatures of 4.0 chromic gut were tied loosely around the nerves [3, 17, 22]. After the operation, the wound was subsequently sutured.

### 2.2. Behavior Assessment

We examined the behavioral signs with mechanical stimulation between 09:00 and 17:00 at one day before (−1) and at 3, 7, 14, 21, and 28 days after CCI to the median nerves of the pre-treatment groups, controls, or uninjured contralateral forepaw. All behavioral measurements were obtained by an investigator blind to the treatment groups.

Mechanical allodynia was estimated by means of Von Frey filaments [24]. Von Frey filaments (Somedic Sales AB, Hörby, Sweden) of different bending forces including 0.145, 0.32, 0.39, 1.1, 1.7, 3.3, 5.1, 8.3, 17, and 24 g were used to test the mechanical threshold of the rat forepaws [17, 22]. Briefly, tests were started with the smallest bending force and continued in increasing order. Each filament was applied five times in the medial surface of a forepaw; the first filament in the series that elicited withdraw three times was regarded as the paw withdrawal threshold. The thresholds of individual rats in each group were averaged and presented as mean and the standard error of the mean (mean ± SEM).

### 2.3. Electrical Stimulation and Electrophysiological Recording

On the 29th day post-CCI operation, the right nerves of the control and CCI rats were reexposed under anesthesia and at least a 12 mm segment proximal to the CCI ligature or on the same level in the control group were isolated. Then, two pairs of platinum hook electrodes were placed on the nerve; the distal pair of the hook electrodes were connected to a Grass S88 stimulator (Grass, Quincy, Mass, USA) for electrical stimulation, and the proximal pair of hook electrodes were connected to an Xction View Data Acquisition System (Model XD-04; Singa, Tao Yuan, Taiwan) for recording. Warm paraffin oil was applied around the exposed nerves to prevent them from drying out. The discharges in the median nerve at pre-electrical stimulation (pre-ES, a 5-min interval just prior to electrical stimulation) and post-ES (a 5-min interval immediately after stimulation) stages were also collected, transformed into frequency histogram (Figure 3), and analyzed by Xction view software (Figure 4) [15]. For electrical stimulation, a 10-min electric square wave pulse current with 0.1 ms duration, 10 Hz frequency, 0.1 mA intensity [15, 17, 22, 25] was applied through a constant current unit.

### 2.4. Tissue Preparation and Immunohistochemistry

Two hours after electrical stimulation (or, in the control group, 2 h after nerve exposure), the rats were reanesthetized and perfused with 500 mL 4% paraformaldehyde in 0.1 M phosphate buffer (PB) at pH 7.4. The brain stems containing the CN were removed, postfixed with the same fixative for 2 hr and stored in PB containing 30% sucrose. The tissue blocks were cut transversely into 30-*μ*m-thick serial sections and orderly divided them into four sets. Two of the four serial sections were treated with 1% H_2_O_2_ and blocked with 5% normal goat serum in 0.1 M PB containing 0.2% Triton X-100 for 2 hr. They were incubated alternatively in rabbit polyclonal anti-NPY (1 : 2000; DiaSorin, Stillwater, Mont, USA) [16, 22, 26] or anti-c-Fos (1 : 2000, Calbiochem, San Diego, Calif, USA) antibodies at 4°C for 48 hr. After several washing with phosphate buffered saline (PBS), the sections were processed with biotinylated anti-rabbit IgG secondary antiserum (Vector, Burlingame, Calif, USA) at room temperature for 2 hr, treated with avidin-biotin-HRP complex (ABC kit, Vector) for 1 hr, and visualized with a Vector SG Substrate Kit. Finally, they were mounted onto gelatinized slides and their images were captured with a digital camera (Nikon, D1X, Tokyo, Japan) through a light microscope (Zeiss, Axiophot, Goettingen, Germany) to measure the NPY-LI fibers or c-Fos-LI cells in the CN.

### 2.5. Data Presentation and Statistical Analysis

All measurements of the behavior tests, rates of injury discharges, NPY-LI fibers, and c-Fos-LI cells in the CN were performed blind to drug treatments. The behavior test of the Von Frey filaments was compared between groups at each time point and statistical analysis was performed with the Student's *t*-test. *P* < 0.05 was considered as significant.

The rate of nerve discharges was presented as the number of discharges divided by the time period of the respective stages and presented as mean ± SEM. In order to investigate the inter-pre-treatment group (Figure 3) and interstage (Table 1) differences, the rates of discharges were compared with two-way ANOVA with a Newman-Keuls posthoc test. *P* < 0.05 was considered statistically significant.

For quantitative analysis, the sections of middle CN, which was defined as an area 0.3–0.7 mm caudal to the obex [16–18, 27], were collected from the entire rostrocaudal extent of the CN. Four sections were collected from the middle region of each animal. To assess the changes in NPY and c-Fos immunoreactivity in the CN, sections were investigated with a Zeiss light microscope and images were captured with a Nikon digital camera at a magnification of 200X. Pictures were processed and evaluated with a computer-based image analysis system (MGDS) and Image Pro-Plus software (Media Cybernetics, Md, USA). The area occupied by NPY-LI fibers and the area of outlined CN were measured [15, 16, 22]. The former divided by the latter was defined as the percentage of area occupied by NPY-LI fibers in the ipsilateral CN and were compared statistically, using two-way ANOVA, with the Newman-Keuls post-test between pretreatment groups (electrically stimulated and nonelectrically stimulated) (Figure 5). The mean number of c-Fos-LI cells in the CN was defined as the number of the surveyed c-Fos-LI cells divided by the number of tissue sections and were calculated and statistically compared with one-way ANOVA and posthoc with the Newman-Keuls test in the respective groups (Figure 7). In order to clarify the relationship between c-Fos-LI cells and the NPY reduction level caused by electrical stimulation (defined as the ratio of the NPY-LI fibers occupied area in the nonstimulated rat minus that in the stimulated rats in different treatment groups), the mean number of c-Fos-LI cells and NPY reduction level of the individual rats were collected and analyzed by linear regression (Figure 8). In order to examine the relationship between c-Fos-LI cells and mechanical allodynia, the mean number of c-Fos-LI cells in the CN and mechanical withdrawal scale (defined as the logarithm of paw withdraw threshold to the base 10 and presented as log_10_-gram) of the individual rats were analyzed by linear regression (Figure 9).

## 3. Results

### 3.1. Effect of Lidocaine Pretreatment on Mechanical Allodynia of the CCI Rats

**Figure 1:**
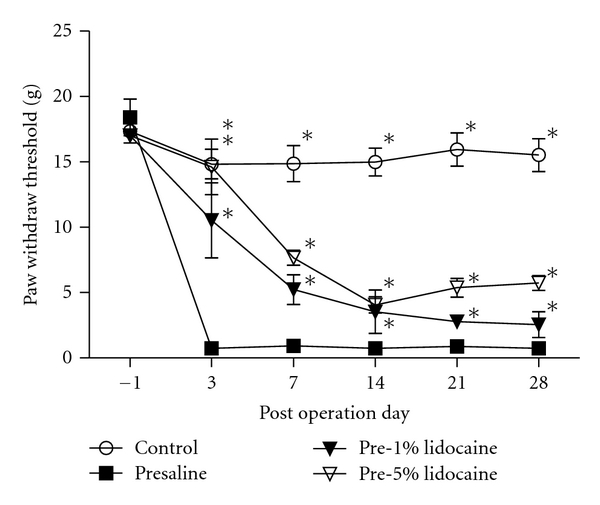
Effects of pre-emptive treatment of saline or various concentrations of lidocaine on paw withdrawal threshold in CCI rats. Pre-treatment of lidocaine increased paw withdrawal threshold and attenuated the TH in CCI rats. *P < 0.05 compared to the presaline group.

Von Frey filament assessment demonstrated that there were no significant differences between bilateral-CCI and unilateral-CCI in mechanical allodynia throughout the experiment period. Von Frey filament tests also showed that in CCI rats, paw withdrawal thresholds decreased from a control of 14.83 ± 1.14 g to 0.75 ± 0.20 g at three days after CCI in the presaline group. Rats established mechanical allodynia three day after CCI, and throughout the 28-day experiment period (control: 15.53 ± 1.26 g, presaline: 0.75 ± 0.20 g, Figure 1). However, pretreatment of lidocaine to CCI increased paw withdrawal threshold and attenuated the tactile hypersensitivity (TH) (Figure 1).

### 3.2. Effect of Lidocaine Pretreatment on Injury Discharges of the Chronic Constriction Injured Median Nerves

**Figure 2:**
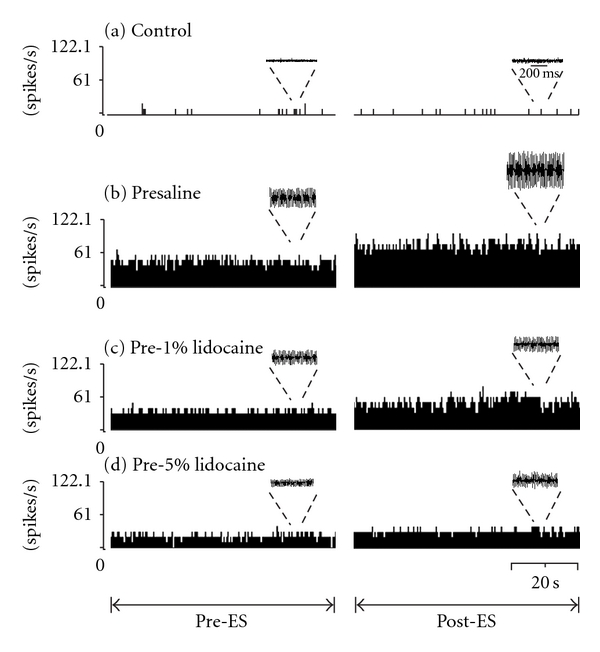
Electrophysiological recording of chronic constricted median nerves at pre- and postelectrical stimulation, (pre-ES and post-ES) stages. Data were collected and transformed into frequency histogram for control (a), presaline (b), pre-1% lido (c), and pre-5% lido (d) groups. Original recordings presented above the histogram, respectively. Note that the rates of discharges were significantly reduced with increasing concentrations of lidocaine pre-treatment.

**Figure 3:**
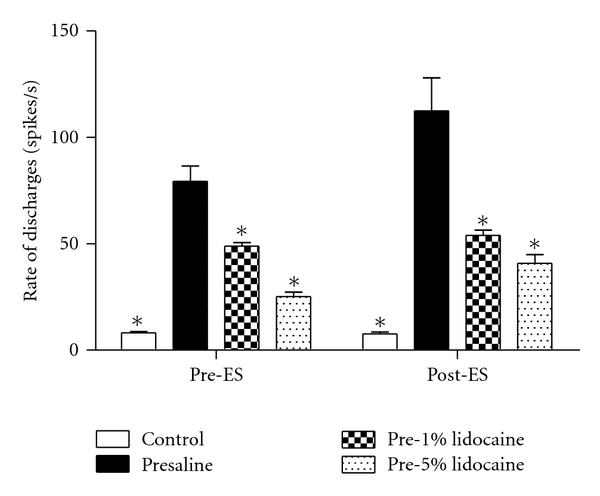
Average rate of discharges (spikes/sec) in different treatment groups at pre-electrical stimulation (pre-ES), and postelectrical stimulation (post-ES) stages. The rates of discharges decreased with increasing doses of lidocaine pre-treatment compared with the presaline group (*P < 0.05 compared with the presaline group).

At 29 days after CCI, electrophysiological recording was used to examine the changes in discharges of the median nerves before and after electrical stimulation (pre-ES and post-ES stages) in all groups (Figure 2). The nerves in the control group displayed a few spikes at pre-ES and post-ES stages (Figure 2(a)). Following median nerve CCI, the rates of discharges at both pre-ES and post-ES stages in all CCI groups increased on the injured nerves (Figures 2(b)–2(d)). Two-way ANOVA of the rate of discharges displayed significant differences between different stages (*F* = 15.09, *P* < 0.05) and between the pretreatment groups (*F* = 47.98, *P* < 0.0001). The rates of discharges at the post-ES stage were significantly higher than that at the pre-ES stage in all CCI groups, respectively (Table 1). Of note, the rates of discharges at both pre-ES and post-ES stages in presaline and pre-1% lido CCI groups were dramatically higher than those in the control group (Figure 3). Furthermore, the rates of discharges in all the lidocaine pretreatment groups at pre-ES (pre-1% lido: 50.31 ± 2.90 Hz, pre-5% lido: 23.76 ± 0.57 Hz) and post-ES (pre-1% lido: 56.86 ± 2.97 Hz, pre-5% lido: 32.86 ± 2.10 Hz) stages were significantly lower than those in the presaline group (pre-ES: 91.86 ± 4.39 Hz; post-ES: 137.39 ± 23.90 Hz), and revealed a dose-dependent suppression manner (Figure 3).

### 3.3. Effect of Lidocaine Pretreatment on NPY and c-Fos Expression in the Cuneate Nucleus

**Figure 4:**
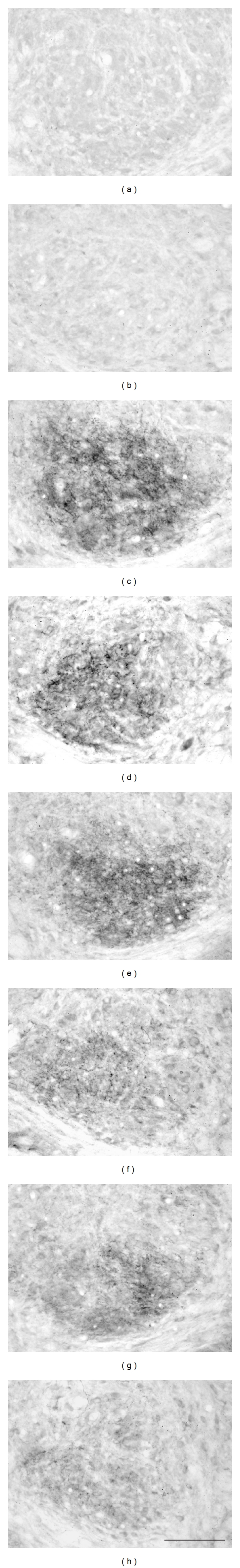
Photomicrographs showing NPY-LI fibers in the middle region of the ipsilateral CN four weeks after chronic constriction injury in control (a, b) or four weeks after CCI without (left panel) or with (right panel) electrical stimulation in presaline (c, d), 1% (e, f), and 5% (g, h) lidocaine pre-treatment groups. Bar = 100 *μ*m.

**Figure 5:**
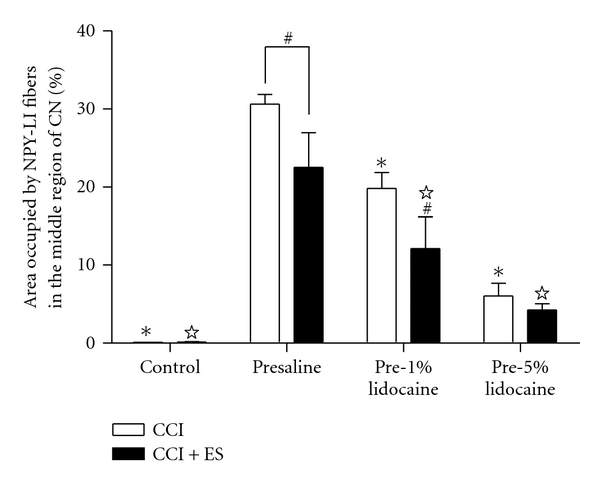
Histograms showing morphometric assessments used to quantify intensity of NPY-LI fibers in the middle CN at four weeks after chronic constriction injury without (CCI) or with electrical stimulation (CCI + ES). It is notable that the intensities of NPY-LI fibers in the CN were significantly lower in rats with electrical stimulation than those without stimulation in the presaline and pre-1% lidocaine groups (^#^ *P* < 0.05 compared with unstimulated side in respective group). The intensity of NPY-LI fibers in the CN was also reduced by lidocaine pretreatment in a dose-dependent manner (*☆*, *P < 0.05 compared with the stimulated and unstimulated side of the presaline group, resp.).

There were little to no NPY-LI fibers in the CN of uninjured control rats, with or without electrical stimulation (control, 0.13 ± 0.01%; control + ES, 0.14 ± 0.03%; Figures 4(a), 4(b), and 5). However, in the presaline group, numerous NPY-LI fibers were detected in the middle CN four weeks after CCI in both the unstimulated (30.62 ± 1.21%) and stimulated (22.53 ± 4.44%) sides (Figures 4(c), 4(d), and 5). The percentage of NPY-LI fibers in the lidocaine pretreatment CCI groups in both the unstimulated (pre-1% lido, 19.81 ± 2.03%; pre-5% lido, 6.04 ± 1.63%) and stimulated (pre-1% lido, 12.12 ± 4.07%; pre-5% lido, 4.26 ± 0.78%) sides of the CN were significantly decreased in a dose-dependent manner compared with those in the presaline group (Figures 4(c)–4(h) and 5). Furthermore, in all CCI groups the amount of NPY-LI fibers in the unstimulated side of the CN (Figures 4(c), 4(e), 4(g), and 5) was significantly higher than that in the stimulated side of the CN (Figures 4(d), 4(f), 4(h), and 5), respectively, except for the pre-5% lido group.

**Figure 6:**
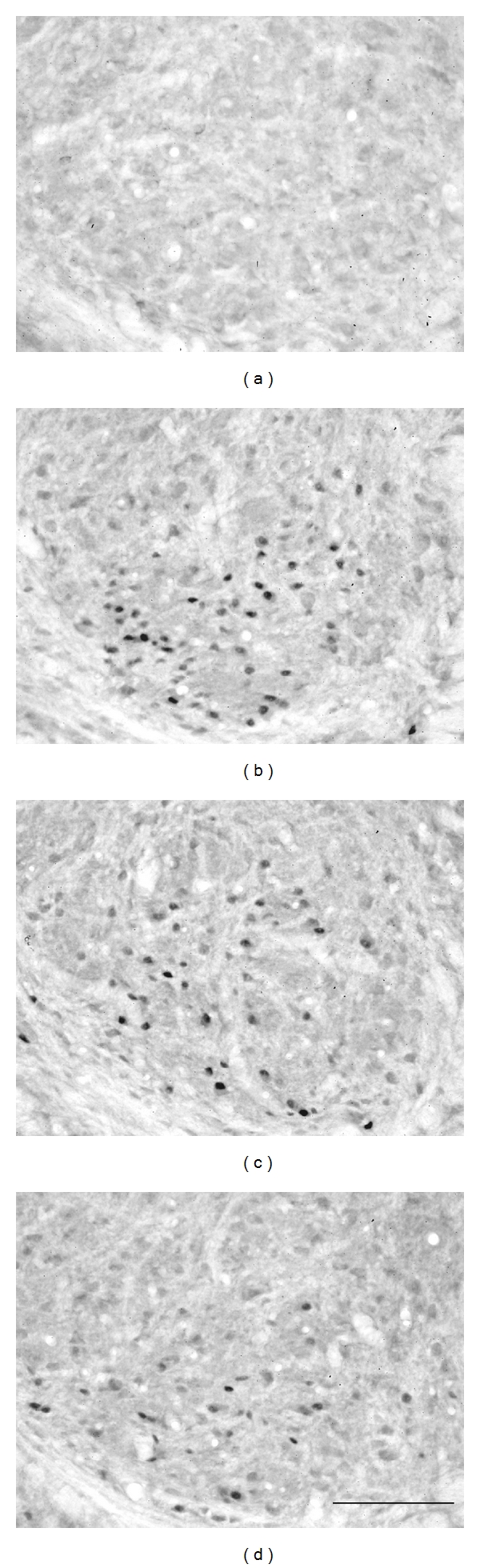
Photomicrographs showing c-Fos-LI cells in the middle region of the CN ipsilateral to electrical stimulation four weeks after chronic constriction injury in control (a), presaline (b), pre-1% lido (c) and pre-5% lido (d) groups. Bar = 100 *μ*m.

**Figure 7:**
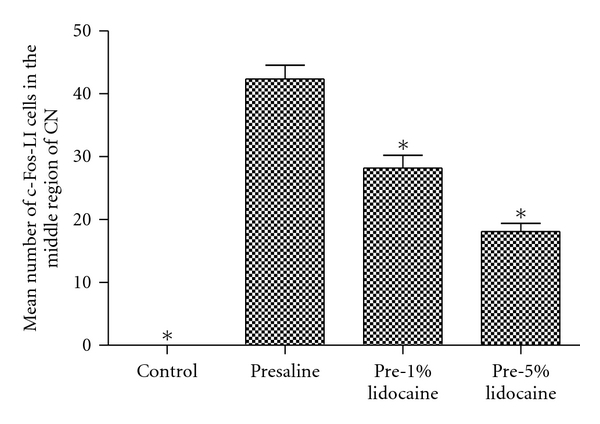
Histograms showing the mean number of c-Fos-LI cells in the middle region of the CN following electrical stimulation in control (a), presaline (b), pre-1% lido (c), and pre-5% lido (d) groups. Note that the mean numbers of c-Fos-LI cells in the pre-1% lido and pre-5% lido groups were significantly less than that in the presaline group (*P < 0.05 compared with the presaline group).

**Figure 8:**
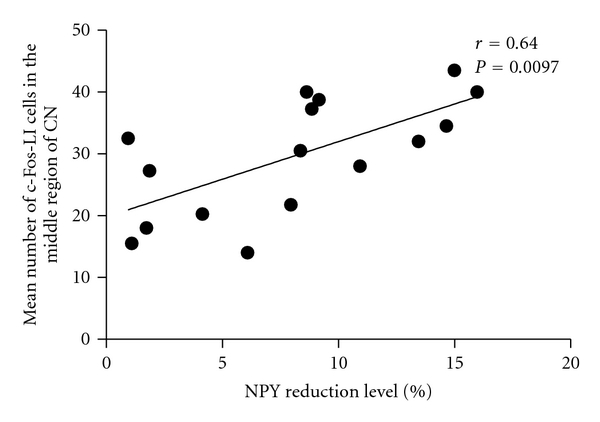
Linear regression of the mean number of c-Fos-LI cells and NPY reduction level in the CN four weeks after chronic constriction injury (each point represents an individual animal; *r* = 0.64, *P* < 0.01).

No, or only very few, c-Fos-LI cells were found in the CN of the control rats, the median nerves with or without electrical stimulation, or in CCI rats without electrical stimulation (Figure 6(a)). However, numerous c-Fos-LI cells were detected when injured median nerves treated with electrical stimulation in all CCI groups and predominantly distributed in the ventral portion of the middle CN (Figure 6). Furthermore, quantitative analysis showed that the mean number of c-Fos-LI cells in the presaline group (42.9 ± 2.8 cells) was significantly greater than that in other groups (Figures 7(b) and 8). The mean number of c-Fos-LI cells in the CN was also reduced by lidocaine pretreatment in a dose-dependent manner (pre-1% lido, 26.6 ± 1.6 cells; pre-5% lido, 18.5 ± 1.4 cells) (Figures 6(c), 6(d), and 7).

**Figure 9:**
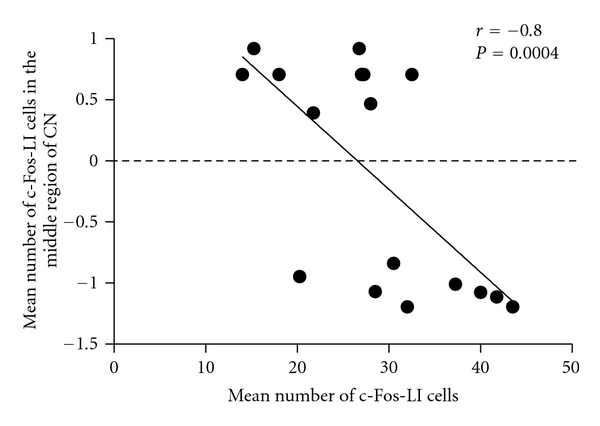
Linear regression of the mean number of c-Fos-LI cells and mechanical withdrawal scale (presented as log_10_-gram) four weeks after chronic constriction injury (each point represents an individual animal; *r* = −0.80, *P* < 0.005).

 In addition, the NPY reduction level was assessed by the percentage of NPY-LI fibers in the stimulated side of the CN subtracted from that in the unstimulated side of the CN, regarded as an index of the extent of NPY release by electrical stimulation. Statistical analysis by linear regression demonstrated that the mean number of c-Fos-LI cells in the stimulated side of the CN was significantly correlated to the NPY reduction level (Figure 8, *r* = 0.64, *P* < 0.01). Finally, linear regression manifested that the mean number of c-Fos-LI cells in the CN was negatively proportional to the mechanical withdrawal scale (Figure 9, *r* = −0.80, *P* < 0.005).

## 4. Discussion

The results of the present study demonstrate the attenuation of TH and reduction of injury discharges following CCI on median nerves by lidocaine pretreatment. Correspondingly, both the level of the injury-induced NPY fibers and the number of injury-induced c-Fos-LI cells in the CN at four weeks after medina nerve CCI were also dose-dependently reduced by lidocaine pretreatment. These results provide a possible mechanism in that the suppression of injury discharges by lidocaine pretreatment not only relieves neuropathic pain but also attenuates the NPY and c-Fos expressions in the CN after CCI.

Following median nerve CCI, signs of mechanical allodynia were detected three days after CCI and lasted throughout the experiment period of 28 days in the present study. Similar results have been reported, where hyperalgesia responses to noxious radiant heat were observed on the second postoperative day and lasted for over two months after sciatic nerve CCI [3]. Another study also indicated that mechanical allodynia was found three to five days following CCI [28], whereas this neuropathic sign was detected one day after CCI in our recent study [22]. There are discrepancies in the time points of neuropathic pain initiating after CCI between various studies. The reason for these discrepancies may be simply due to different time points being examined. We focused on the role of lidocaine pretreatment in the paw withdrawal threshold of the CCI rats. The reductions in the paw withdrawal threshold after median nerve CCI were reversed by lidocaine pretreatment in a dose-dependent manner. A previous study [6] reported that lidocaine pretreatment relieved thermal hyperalgesia for a long postoperative period (up to three weeks) after sciatic nerve CCI. However, another study showed that prior to spinal nerve ligation (SNL), lignocaine pretreatment increased the paw withdrawal threshold for only 24 h [29]. These discrepancies may be related to differences in the injury models used in the above-mentioned studies (CCI versus SNL). The lesion site to the DRG in the CCI model was more distal than that in the SNL model. The injury level caused by the former was less severe than that by the latter, so the neuropathic pain induced by the CCI model could be prevented by lidocaine pre-treatment.

Furthermore, four weeks after CCI (29 days postinjury), a significant increase in the number of spikes in all CCI groups, which was regarded as ectopic discharges induced after nerve injury. Then, our results also demonstrated that the ectopic discharges were suppressed by lidocaine pre-treatment in a dose-dependent manner. The suppression in ectopic discharges was considered as one of the contributing factors in relieving neuropathic pain induced by median nerve CCI. Our recent study reported that ectopic discharges evoked by median nerve transection (MNT) were suppressed by pre-treatment with 5% and 10% lidocaine, but not 1% lidocaine [15]. However, in the present study, ectopic discharges induced by CCI were significantly attenuated by 1% lidocaine pre-treatment. This discrepancy may also be explained by the difference in injury model employed between these two studies. The injury severity induced by CCI was milder than that by MNT. For this reason, the rate of injury discharges in the CCI rats, but not MNT, could be significantly reduced by low-dose (1%) lidocaine pre-treatment.

The present study further demonstrated that a significant increase in NPY in the CN at four weeks (29 days) post-CCI was also dose-dependently reduced by lidocaine pre-treatment. Previous studies have reported that intense afferent discharges and depolarization enhanced NPY induction [30, 31]. Furthermore, the NPY induction in the CN exclusively originated from injured DRG neurons, particularly medium- and large-size neurons, via primary afferent fibers [26]. It is reasonable to infer that the reduction of NPY expression in the CN would result from the suppression in injury discharges following CCI with lidocaine pre-treatment. This is consistent with NPY reduction in the CN after MNT [15] with lidocaine pre-treatment and in the spinal cord laminae 3-4 following sciatic nerve CCI with MK-801 and clonidine pre-treatment [30]. Taken together, these findings suggest that the magnitude of nerve discharges may be one of the most important factors to induce NPY synthesis.

In the rats with bilateral median nerve CCI, c-Fos-LI cells were found only in the CN with electrical stimulation, but not in the unstimulated side of the CN; the level of NPY-LI fibers in the stimulated side of the CN was also significantly lower than that in the unstimulated side. One possible explanation for this is that NPY is released from the injured median nerve in the stimulated side of the CN resulting in NPY reduction and induced c-Fos expression in the same region. This is compatible with previous studies where NPY reduction and c-Fos induction were detected in the stimulated side of the CN following electrical stimulation with the transected median nerve; injection of an NPY receptor antagonist into the CN coupled with electrical stimulation to the injured nerve resulted in a dramatic decrease in the number of c-Fos-LI cells in the ipsilateral CN [15, 22]. In the present study, we also found that the number of c-Fos-LI cells in the CN after electrical stimulation of the injured nerve was dose-dependently reduced by lidocaine pre-treatment. Statistical analysis further demonstrated that the number of c-Fos-LI cells in the stimulated side of the CN was significantly correlated to the level of NPY reduction. Taken together, these results suggest that the amount of NPY release (NPY reduction level), following electrical stimulation of the injured nerve, directly modulates c-Fos expression in the CN.

Although the function of c-Fos induction in the CN remains uncertain, the expression of c-Fos immunoreactivity in the spinal cord has been considered as a convincing marker of pain [19, 20]. Our results showed that the number of c-Fos-LI cells in the CN coincided with the reduction in paw withdrawal thresholds, regarded as mechanical allodynia. This is in agreement with a previous study which reported that the number of c-Fos-LI cells was positively associated with the magnitude of mechanical allodynia [22]. Earlier studies have also clarified that after electrical stimulation of the injured median nerve, about 78% of c-Fos-LI cells in the middle CN were cuneothalamic projection neurons (CTNs) [17, 18]. This study further showed that the number of c-Fos-LI cells was dose-dependently reduced by lidocaine pre-treatment. Injury discharges have been reported to be implicated in the increase of c-Fos LI cell expression in the spinal cord dorsal horn [32–34], while lidocaine pre-treatment attenuates the discharges to prevent c-Fos induction [21, 34].

## 5. Conclusions

Our results suggest that lidocaine pre-treatment dose-dependently suppressed injury discharges development to attenuate NPY expression after median nerve injury. This in turn significantly reduces the NPY release to decrease the transmitting of TH to the thalamus and c-Fos expression in the CTNs.

## Figures and Tables

**Table 1:** Statistical comparison of mean values of the nerve discharges.

Group	Pre-ES	Post-ES
Control	8.21 ± 0.48 Hz	7.65 ± 0.95 Hz
Presaline	91.86 ± 4.39 Hz	137.39 ± 23.90 Hz*
Pre-1% lido	50.31 ± 2.90 Hz	56.86 ± 2.97 Hz*
Pre-5% lido	23.76 ± 0.57 Hz	32.86 ± 2.10 Hz*

Control: sham operation with electrophysiological recording at the corresponding stages. ES: electrical stimulation. Data are presented as mean ± standard error of the mean (SEM) and *P < 0.05 compared with the pre-ES stage.
